# Control of Toll-like Receptor-mediated T Cell-independent Type 1 Antibody Responses by the Inducible Nuclear Protein IκB-ζ*

**DOI:** 10.1074/jbc.M114.553230

**Published:** 2014-08-14

**Authors:** Fumito Hanihara-Tatsuzawa, Hanae Miura, Shuhei Kobayashi, Takayuki Isagawa, Atsushi Okuma, Ichiro Manabe, Takashi MaruYama

**Affiliations:** From the ‡ Laboratory of Cell Recognition and Response, Department of Developmental Biology and Neurosciences, Graduate School of Life Sciences, Tohoku University, Sendai, Miyagi 980-8578,; the § Department of Genomic Pathology, Medical Research Institute, Tokyo Medical and Dental University, Tokyo 113-8510,; the ¶ Department of Cardiovascular Medicine, Graduate School of Medicine, University of Tokyo, Tokyo 113-8655, and; the ‖ Laboratory of Cell Signaling, School of Medicine, Gifu University, Gifu 501-1194, Japan

**Keywords:** Cell Differentiation, Cellular Immune Response, Immunology, Toll-like Receptor (TLR), Transcription Factor, B Cell, IκB-z, Activation-induced Cytidine Deaminase (AID)

## Abstract

Antibody responses have been classified as being either T cell-dependent or T cell-independent (TI). TI antibody responses are further classified as being either type 1 (TI-1) or type 2 (TI-2), depending on their requirement for B cell-mediated antigen receptor signaling. Although the mechanistic basis of antibody responses has been studied extensively, it remains unclear whether different antibody responses share similarities in their transcriptional regulation. Here, we show that mice deficient in IκB-ζ, specifically in their B cells, have impaired TI-1 antibody responses but normal T cell-dependent and TI-2 antibody responses. The absence of IκB-ζ in B cells also impaired proliferation triggered by Toll-like receptor (TLR) activation, plasma cell differentiation, and class switch recombination (CSR). Mechanistically, IκB-ζ-deficient B cells could not induce TLR-mediated induction of activation-induced cytidine deaminase (AID), a class-switch DNA recombinase. Retroviral transduction of AID in IκB-ζ-deficient B cells restored CSR activity. Furthermore, acetylation of histone H3 in the vicinity of the transcription start site of the gene that encodes AID was reduced in IκB-ζ-deficient B cells relative to IκB-ζ-expressing B cells. These results indicate that IκB-ζ regulates TLR-mediated CSR by inducing AID. Moreover, IκB-ζ defines differences in the transcriptional regulation of different antibody responses.

## Teaser

**Background:** IκB-ζ, a member of the IκB family of nuclear proteins that regulates transcription, can be induced by Toll-like receptor (TLR) signaling.

**Results:** IκB-ζ deficiency in B cells reduced T cell-independent antibody response-1.

**Conclusion:** IκB-ζ is a key regulator of TLR-mediated class switch recombination (CSR) in B cells.

**Significance:** T cell-dependent and -independent antibody responses are regulated by different mechanisms.

## Introduction

Antibody responses are central to protecting hosts from pathogen infection. After B cells recognize antigens, they initiate three steps, proliferation, class switch recombination (CSR),^2^ and plasma cell differentiation, that are required for antibody production. In terms of antibody responses, antigens are typically classified as being either T cell-dependent (TD) or T cell-independent (TI) antigens (1). TD antigens are soluble proteins or peptides that are recognized by specific B cell receptors and induce clonal activation of B cells; TD antibody responses require the interaction of the CD40 ligand on a T cell with a CD40 receptor on a B cell (2, 3). In contrast, TI antigens can initiate antibody responses independently of T cells. TI antibody responses are classically defined as TI type 1 (TI-1) antigens and TI type 2 (TI-2) antigens, depending on their requirement for Btk, which is a key kinase needed for B cell antigen receptor (BCR) signaling (4, 5). The TI-1 antigen TNP-LPS, but not the TI-2 antigen TNP-Ficoll, can elicit anti-TNP plaque-forming cell responses in Btk-deficient mice (4). Thus, BCR signaling is necessary for responses triggered by TI-2 antigen but dispensable for responses triggered by the TI-1 antigen. TI-2 antigens, which contain a repetitive epitope such as capsular polysaccharide, induce strong BCR signaling by engaging multiple BCRs, which induces clonal B cell activation and antigen-specific immunoglobulin (Ig) production (6). TI-1 antigens, such as LPS, are considered to act as mitogens that stimulate B cells to produce polyclonal antibodies following Toll-like receptor (TLR) stimulation (7, 8). However, the polysaccharide moiety of the LPS binds to the BCRs of multiple B cells (9). As a consequence, LPS can induce the production of not only polyclonal Igs but also antigen-specific Igs by co-engaging TLR4 and BCR. In addition, co-stimulation of other TLR ligands and BCR induces strong activation-induced cytidine deaminase (AID) expression and a high rate of CSR. Thus, TLR-mediated antibody responses are divided into BCR-independent polyclonal responses and BCR-dependent clonal responses.

Although the mechanism of antibody responses varies widely between the types of antigens described above, it remains unclear whether common transcriptional factors regulate both TD and TI antibody responses. CSR in B cells switches one isotype of an antibody to another. AID is thought to be a master regulator of CSR, which is regulated by transcriptional factors that include Bach2, IRF4, and Hoxc4 (10–16).

The observation that deficiencies in any of these four transcriptional factors impair both TD- and TI-induced AID and CSR suggests that the same mechanisms of transcriptional regulation operate both in TD and TI antibody responses. However, the signaling pathway activated by CD40, which is a key receptor for the TD antibody response, clearly differs from that triggered by TLR activation. Thus, it is possible that transcriptional regulation of AID is regulated by factors that differ between the TD and TI antibody responses.

This study focused on the function of nuclear IκB family member IκB-ζ in B cell-mediated antibody responses. IκB-ζ is a transcriptional regulator that interacts with NF-κB in macrophages (17, 18). Previous studies showed that IκB-ζ is key regulator of innate and adaptive immune responses, such as Th17 development, NK cell-derived IFN-γ production, and IL-6 production in macrophages (19–22). In epithelial cells, a deficiency in IκB-ζ causes apoptosis, which induces Sjögren's syndrome-like inflammation (23). We have recently shown that IκB-ζ controls TLR-induced IL-10 production in B cells (24). However, a role for IκB-ζ in B cell antibody responses has never been reported. Here, we report that a deficiency of IκB-ζ specifically in B cells impaired TI-1, but not TD and TI-2, antibody responses both *in vitro* and *in vivo*. Furthermore, we showed that a deficiency in IκB-ζ-impaired TLR induced proliferation, CSR, and differentiation of plasma cells. Notably, IκB-ζ-deficient B cells did exhibit AID expression by anti-CD40 stimulation but not LPS stimulation. Furthermore, IκB-ζ is essential for the co-stimulation of either TLR2 or TLR9 with BCR to ensure CSR. These findings indicate that the IκB-ζ-regulated transcriptional network controls TLR-mediated antibody responses. These results reveal that IκB-ζ defines a key distinction between TD and TI antibody responses.

## EXPERIMENTAL PROCEDURES

### 

#### 

##### Mice

The loxP-flanked *Nfkbiz* allele has been described previously (23). We generated Nfkbiz *^fl^*^/Δ^ Mb1^cre/+^ mice by crossing of Nfkbiz *^fl^*^/Δ^ mice with Mb-1 cre mice (25). All mice were kept under specific pathogen-free conditions in the animal facilities of Tohoku University. All animal protocols were approved by the Institutional Animal Care and Use Committee.

##### Cells

B cells were purified from the spleen by using a B cell isolation kit for negative depletion of cells that express CD43, CD4, or Ter-119 (Miltenyi Biotech, Bergisch Gladbach, Germany). Use of the kit according to the manufacturer's protocol resulted in a purity of >95% of B220^+^ B cells. The murine B lymphoma cell line CH12F3-2A (Riken Cell Bank, Tsukuba, Japan) was cultured in RPMI 1640 medium supplemented with 10% heat-inactivated FCS, 100 units/ml penicillin, 100 μg/ml streptomycin, 2 mm l-glutamine, 0.1 mm nonessential amino acids, 1 mm sodium pyruvate, and 50 μm 2-mercaptoethanol at 37 °C in 5% CO_2_.

##### Reagents and Antibodies

LPS from *Escherichia coli* O111:B4 was purchased from List Biological Laboratories Inc. (Campbell, CA). A phosphorothioate-stabilized CpG oligodeoxynucleotide (ODN1826, 5′-TCCATGACGTTCCTGACGTT-3′) was synthesized by Sigma Genosys. *S*-[2,3-Bis(palmitoyloxy)-(2-*RS*)-propyl]-*N*-palmitoyl-(*R*)-Cys-(*S*)-Ser-(*S*)-Lys_4_-OH (Pam_3_CSK_4_) was synthesized by Peptide Institute, Inc. (Osaka, Japan). 2,4,6-Trinitrophenyl (TNP)-keyhole limpet hemocyanin (KLH), TNP-aminoethylcarboxymethyl-Ficoll, and TNP-LPS were purchased from Biosearch Technologies (Petaluma, CA). Carboxyfluorescein succinimidyl ester (CFSE) was purchased from eBioscience Inc. (San Diego, CA). 4′,6-Diamidino-2-phenylindole (DAPI) was purchased from Dojindo (Kumamoto, Japan). Commercially available antibodies used in the study are shown in Table 1. Anti-IκB-ζ monoclonal antibodies were raised against a bacterially expressed recombinant mouse IκB-ζ protein injected into rats (21).

**TABLE 1:** **Commercially available antibodies used in this study** The following abbreviations are used: IB, immunoblotting; PE, phycoerythrin; FC, flow cytometry.

Antigen	Clone	Conjugate	Vendor	Application
β-Actin	AC-15	HRP	Abcam	IB
B220	RA3-6B2	PE	BioLegend	FC
B220	RA3-6B2	SPRD	Beckman Coulter	FC
CD3	17A2	Alexa Fluor® 488	BioLegend	FC
CD11b	M1/70	APC	eBioscience	FC
CD11c	N418	PE	BioLegend	FC
CD93	AA4.1	PE	BioLegend	FC
IgM	eB121-15F9	FITC	eBioscience	FC
IgD	11-26	Alexa Fluor® 647	eBioscience	FC
IgG1	RMG1-1	APC	BioLegend	FC
IgG3	R40–82	FITC	BD Biosciences	FC
CD21	7G6	FITC	BD Biosciences	FC
CD23	B3B4	Alexa Fluor® 647	BioLegend	FC
CD138	MI15	FITC	BD Biosciences	FC
CD16/32	93		BioLegend	FC (blocking)
IgG (rabbit)			Cell Signaling	ChIP
Acetyl-histone H3 (Lys-27)	D5E4		Cell Signaling	ChIP
IgG (rabbit)		HRP	Chemicon	IB
IgM (goat, F(ab′)_2_)			Jackson ImmunoResearch	Ligand
Anti-IgD-Dex		Dextran	Fina Biosolutions	Ligand
Anti-CD40	1C10		eBioscience	Ligand

##### Plasmids

The pGL4.12-SV40-[luc2CP]-Nfkbiz-3′-UTR reporter plasmid for IκB-ζ post-transcriptional regulation was described previously (26). Reporter plasmids for AID regulatory elements were amplified by PCR-mediated amplification of genomic clones that contain *Aicda*, the gene that encodes AID. The amplified minimal region (−101 to +1), region 1 (−1500 to +101), and region 4 (−9224 to −7424) were each cloned separately into the pGL3-basic vector (Promega) upstream of the firefly luciferase-encoding region. In contrast, region 2 (+121 to +2221) and region 3 (+16,278 to +18,378) were each cloned separately into the pGL3-basic vector downstream of the luciferase sequence. pcDNA3 and phRL-TK were obtained from Invitrogen and Promega Corp. (Madison, WI), respectively. Expression vectors for FLAG-tagged mouse IκB-ζ and p65 overexpression were constructed as described previously (21).

##### Immunization and ELISA

Basal serum Ig titers were quantified by ELISA by using HRP-conjugated Ig from Southern Biotech (Victoria, Australia). To evaluate T cell-dependent or T cell-independent antibody responses, mice were administered intraperitoneal doses of the following: 100 μg of TNP-KLH in alum; 50 μg of TNP-Ficoll; or 50 μg of TNP-LPS. Titers of antibodies to TNP were measured by ELISA with plate-bound TNP-conjugated BSA (Biosearch Technologies) and isotype-specific horseradish peroxidase-conjugated secondary antibodies (Southern Biotech).

##### Flow Cytometry

Cell surface antigens were stained in the dark at 4 °C with antibodies diluted in PBS that contained 0.5% bovine serum albumin. Cells were analyzed using a Galios instrument (Beckman Coulter). Dead cells (DAPI^+^) were excluded from the analysis. B cells, T cells, dendritic cells, and macrophages with the B220^+^, CD3^+^, CD11c^+^, or CD11b^+^ genotype were purified (>95%) from Nfkbiz*^fl^*^/Δ^ or Nfkbiz*^fl^*^/Δ^ Mb1^cre/+^ mice using a Aria II cell sorter (BD Biosciences).

##### Analysis of in Vitro CSR

Splenic B cells were stimulated with LPS (20 μg/ml) or anti-CD40 antibodies and the additional reagents indicated below. No additional reagents were added for CSR to IgG3 and IgG2b, but 5 ng/ml mouse IL-4 (5 ng/ml) was added for CSR to IgG1, 50 ng/ml mouse IFN-γ (PeproTech) was added for CSR to IgG2a, and 1 ng/ml TGF-β1 (PeproTech) was added for CSR to IgA. Supernatants from cell cultures were collected on day 7 to analyze the secretion of Igs. To analyze surface Igs, cells were collected on day 3 and stained with phycorerythrin-labeled rat mAb to mouse IgG1.

##### Real Time RT-PCR

Total RNA was prepared using RNAiso Plus. Levels of mRNA were quantified by real time RT-PCR using the High Capacity cDNA reverse transcription kit (Applied Biosystems, Foster City, CA) and SYBR Premix EX TaqII (Takara Bio Inc., Otsu, Japan) with a LightCycler 3302 instrument (Roche Diagnostics). The primer sequences used are shown in Table 2.

**TABLE 2:** **Oligonucleotide primers used in the study**

Gene	Orientation	Sequence
*Blimp-1*	Forward	5′-TTCTCTTGGAAAAACGTGTGGG-3′
	Reverse	5′-GGAGCCGGAGCTAGACTTG-3′
*Aicda*	Forward	5′-CGTGGTGAAGAGGAGAGATAGTG-3′
	Reverse	5′-CAGTCTGAGATGTAGCGTAGGAA-3′
*CD79b*	Forward	5′-CCACACTGGTGCTGTCTTCC-3′
	Reverse	5′-GGGCTTCCTTGGAAATTCAG-3′
*Gapdh*	Forward	5′-TGTGAACGGATTTGGCT-3′
	reverse	5′-AAGACGCCAGTAGACTC-3′
*Nfkbiz*	Forward	5′-TCTCACTTCGTGACATCACC-3′
	reverse	5′-GGTTGGTATTTCTGAGGTGGAG-3′
I_μ_-C_γ_1	Forward	5′-GGCCCTTCCAGATCTTTGAG-3′
	Reverse	5′-ATGGAGTTAGTTTGGGCAGCA-3′
I_μ_-C_γ_1	Forward	5′-ACCTGGGAATGTATGGTTGTGGCTT-3′
	Reverse	5′-ATGGAGTTAGTTTGGGCAGCA-3′
*Aicda* −8.5 kb	Forward	5′-TGGCTTTTCATACCCCAGAG-3′
	Reverse	5′-TGGTTGTTGGATTGCTTCAA-3′
*Aicda* −1.5 kb	Forward	5′-GGCCAAAGTAGGGCAAAGG-3′
	Reverse	5′-AGGTGGTGGGTGGACAAGTC-3′
*Aicda* TSS	Forward	5′-CACACAACAGCACTGAAGCA-3′
	Reverse	5′-ATATCGGTCTCCAGCGTGAC-3′
*Aicda* +0.2 kb	Forward	5′-CCCTCTGCTCAGGTCTTTTG-3′
	Reverse	5′-CAGGACAAGTCAAGGCTTCC-3′
*Aicda* +17 kb	Forward	5′-CAGCTGTATTTGTTTGTTCTTTAGTAATTG-3′
	Reverse	5′-CATCCCGAAACACATATACTCACTTT-3′
*Blimp1* promoter	Forward	5′-CATCGCGGCGGCTGGTAGGAGTG-3′
	Reverse	5′-TGTCTGTGCGAGCGAGCGAGTGA-3′

##### RNA Sequence

Total RNA were purified from LPS-stimulated (20 μg/ml) splenic B cells on day 3 by RNeasy (Qiagen, Venlo, Netherlands), according to this study. Poly(A) mRNAs were purified from total RNA using the poly(A) mRNA magnetic isolation module (New England Biolabs, Ipswich, MA). Libraries were prepared using the Next Ultra RNA library prep kit for Illumina (New England Biolabs). After the preparation of the RNA library, we performed sequencing using an Illumina IIx genome analyzer. Reads (38 bp) were mapped to the mouse genome (mm9 from University of California at Santa Cruz genome browser database) using the TopHat Version 2.0.0 algorithm with default settings. Only reads with a Phred quality score greater than or equal to 25 were analyzed. The BED Tools package (27) was used to filter rRNA (ribosomal RNA) and tRNA (transfer RNA), with rRNA and tRNA annotations downloaded from the University of California at Santa Cruz table browser. The data have been entered into the NCBI Gene Expression Omnibus (accession number GSE57837). The data were modified and shown in Table 3. (In order to exclude those genes with very low expression, only genes with a RNA-seq score of >0.05 in at least one sample were chosen. Of this gene set, those genes with a <0.5-fold change in expression in the sample from B-cell-specific Nfkbiz-deficient (cKO) mice compared to the sample from control are shown.)

**TABLE 3:** **RNA sequence data**

	GeneID	Control_1	Control_2	cKO_1	cKO_2	Symbol
1	100504746	0.00992	0.00861	0.00023	0.00037	LOC100504746
2	11989	0.00988	0.006	0.00041	0.00057	Slc7a3
3	407828	0.00624	0.00575	0.00041	0.0004	BC023969
4	99899	0.01149	0.00435	0.00075	0.00064	Ifi44
5	14468	0.01154	0.01038	0.00087	0.00141	Gbp1
6	14968	0.00008	1.14951	0	0	H2-Ea-ps
7	12142	0.00835	0.00458	0.00083	0.00075	Prdm1
8	11628	0.00702	0.00365	0.0005	0.00086	Aicda
9	80879	0.00622	0.00712	0.00114	0.0007	Slc16a3
10	12578	0.04074	0.02131	0.00431	0.00526	Cdkn2a
11	14255	0.01311	0.0055	0.00101	0.002	Flt3
12	14469	0.07122	0.0465	0.00799	0.01241	Gbp2
13	17687	0.00099	0.01794	0.00054	0.00108	Msh5
14	433003	0.0155	0.00047	0.00057	0.00049	Gm5481
15	100503322	0.00506	0.00099	0.0005	0.00046	LOC100503322
16	665298	0.01644	0.20552	0.01244	0.01481	Gm11942
17	15130	0.00638	0.00116	0.00074	0.00056	Hbb-b2
18	11910	0.00688	0.00441	0.00133	0.00143	Atf3
19	67620	0.01066	0.01645	0.00388	0.003	Lrp2bp
20	70377	0.00935	0.00573	0.00171	0.00216	Derl3
21	654824	0.01971	0.03366	0.00745	0.00648	Ankrd37
22	15129	0.00657	0.00113	0.00086	0.0007	Hbb-b1
23	229900	0.01559	0.01038	0.00314	0.00434	Gbp6
24	64214	0.00596	0.00578	0.00156	0.00205	Rgs18
25	13401	0.005	0.0042	0.0017	0.00116	Dmwd
26	55932	0.05787	0.04438	0.01307	0.01858	Gbp3
27	231932	0.03635	0.02884	0.00954	0.01108	Gimap7
28	78376	0.00613	0.02546	0.00299	0.00557	Ng23
29	11676	0.01473	0.01698	0.00488	0.00555	Aldoc
30	59289	0.00714	0.00489	0.00176	0.00217	Ccbp2
31	14990	0.00237	0.01556	0.00189	0.00215	H2-M2
32	171543	0.01357	0.01533	0.00523	0.0044	Bmf
33	675325	0.00388	0.02543	0.00358	0.00329	2410017I17Rik
34	27053	0.00534	0.00272	0.00153	0.00119	Asns
35	229898	0.02046	0.01421	0.00545	0.00684	Gbp5
36	106572	0.01084	0.01372	0.00495	0.00386	Rab31
37	100039192	0.02238	0.02113	0.00814	0.00766	Gm10395
38	100039257	0.02238	0.02113	0.00814	0.00766	Gm9746
39	100503205	0.0057	0.00243	0.00128	0.00146	LOC100503205
40	98388	0.02482	0.0147	0.00642	0.008	Chst10
41	58206	0.07413	0.04684	0.01863	0.02696	Zbtb32
42	66039	0.02275	0.02158	0.0086	0.00826	D14Ertd449e
43	27762	0.00526	0.02189	0.00302	0.00559	D17H6S56E-3
44	14190	0.0063	0.00391	0.0016	0.00231	Fgl2
45	17855	0.01736	0.01724	0.0058	0.00779	Mvk
46	229905	0.00754	0.00639	0.00208	0.00361	Ccbl2
47	667618	0.01432	0.08527	0.01321	0.01477	Gm8730
48	20720	0.01479	0.01247	0.00582	0.00527	Serpine2
49	20530	0.01297	0.00992	0.00442	0.00489	Slc31a2
50	16153	0.00622	0.00357	0.0021	0.00178	Il10
51	16443	0.00984	0.00949	0.00372	0.00429	Itsn1
52	107993	0.01225	0.00948	0.00401	0.00507	Bfsp2
53	19011	0.00657	0.0081	0.00291	0.00327	Endou
54	68099	0.0051	0.00195	0.00103	0.00179	Fam92a
55	100504270	0.16903	0.18303	0.07245	0.07966	LOC100504270
56	27208	0.00543	0.00661	0.00122	0.00554	Snord33
57	67657	0.01426	0.01375	0.00607	0.00618	Rabl3
58	56742	0.02519	0.01922	0.00916	0.0102	Psrc1
59	12176	0.00671	0.00876	0.00298	0.00386	Bnip3
60	106389	0.01417	0.01049	0.00528	0.00553	Eaf2
61	244418	0.0099	0.01528	0.00525	0.00574	D8Ertd82e
62	21991	0.21382	0.2287	0.08767	0.11258	Tpi1
63	110196	0.18026	0.1419	0.05971	0.08702	Fdps
64	74953	0.02659	0.02749	0.00836	0.01781	4930483K19Rik
65	66995	0.02521	0.02191	0.01038	0.01087	Zcchc18
66	14085	0.01988	0.01927	0.008	0.01001	Fah
67	56473	0.02772	0.02727	0.01037	0.01529	Fads2
68	77252	0.01003	0.00645	0.00325	0.00422	9430038I01Rik
69	27218	0.01127	0.00988	0.00443	0.00541	Slamf1
70	12491	0.18182	0.15953	0.06727	0.09321	Cd36
71	12569	0.01317	0.01056	0.00477	0.00637	Cdk5r1
72	16909	0.11698	0.09582	0.04946	0.04977	Lmo2
73	67724	0.01524	0.01319	0.00669	0.00674	Pop1
74	100502995	0.13326	0.12199	0.05511	0.06676	LOC100502995
75	212508	0.01067	0.0078	0.00413	0.00461	Mtg1
76	64657	0.01677	0.02286	0.00762	0.01157	Mrps10
77	20439	0.0935	0.07385	0.0356	0.04478	Siah2
78	18194	0.02836	0.02712	0.01296	0.01431	Nsdhl
79	18968	0.02528	0.02123	0.01157	0.01135	Pola1
80	68603	0.04139	0.04015	0.01773	0.02294	Pmvk
81	17064	0.00882	0.00801	0.00435	0.00399	Cd93
82	231070	0.07453	0.0702	0.03283	0.03936	Insig1
83	20250	0.2147	0.19924	0.09324	0.11346	Scd2
84	232406	0.01562	0.01282	0.00564	0.00884	BC035044

##### Immunoblotting

Cells were lysed, subjected to 10% SDS-PAGE, and analyzed by immunoblotting with anti-IκB-ζ or anti-β-actin antibodies, and secondary antibodies were conjugated with horseradish peroxidase. Bound antibodies were visualized by chemiluminescence after incubation with Immobilon Western Chemiluminescent HRP substrate.

##### Retroviral Transduction

The cDNAs that encode BATF or AID were cloned into pMY-IREIS-EGFP (28). Recombinant retroviruses were prepared by transfecting the Plat-E packaging cells with plasmid, using calcium phosphate transfection. B cells were stimulated with anti-IgD for 24 h and were infected with the viral supernatants in the presence of Polybrene (5 μg/ml) by spin infection for 90 min at 800 × *g* at 32 °C. The cells were incubated at 37 °C in 5% CO_2_ for 2 h and stimulated by exposure to both LPS and IL-4 to induce CSR.

##### Transfection

CH12F3-2A cells were transfected by electroporation with each reporter plus phRL-TK (Promega Corp., Madison, WI). One day after electroporation, the cells were stimulated either with LPS plus IL-4 or with anti-CD40 plus IL-4.

##### Luciferase Assay

Cells were stimulated as indicated and lysed for luciferase assay. Luciferase activity was measured by the Dual-Luciferase^TM^ reporter assay system according to the manufacturer's instructions (Promega Corp.).

##### ChIP Assay

Splenic B cells were activated with LPS plus IL-4 for 3 days. Cells were fixed for 10 min at 25 °C in 1% (w/v) formaldehyde. Cross-linking was terminated by the addition of 150 mm glycine. After being washed with ice-cold PBS containing 0.5% BSA, cells were lysed by sonication in SDS lysis buffer (1% (w/v) SDS, 10 mm EDTA, and 50 mm Tris, pH 8.0). Debris was removed by centrifugation. Lysates were cleared by mixing with Protein G-Sepharose (GE Healthcare) plus salmon sperm DNA (Invitrogen). A ChIP assay was performed using antibodies against acetyl-histone H3 (Lys-27) and normal rabbit IgG. Quantitative PCR was performed with a LightCycler using the primers described in Table 2.

##### Statistical Analysis

Paired data were evaluated with Student's *t* test. A value of *p* < 0.05 was considered statistically significant.

## RESULTS

### 

#### 

##### Mice Deficient in IκB-ζ Specifically in Their B Cells Have Impaired TI-1 Antibody Responses

**FIGURE 1.**
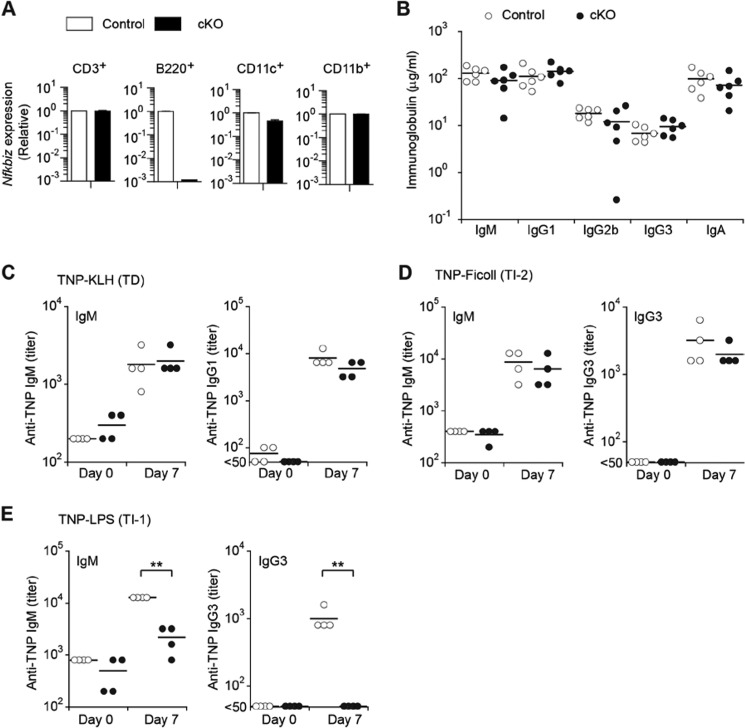
**Mice deficient in IκB-ζ specifically in their B cells exhibit impaired T-independent type 1 immune responses.** *A,* relative levels of expression of *Nfkbiz* mRNA in splenic T cells, B cells, dendritic cells, and macrophage from control and cKO mice. The *Nfkbiz*/*Gapdh* ratio in control cells was arbitrarily set as “1.” Data shown are the mean ± S.D. of a duplicate sample. *B,* immunoglobulin titers in sera of control and cKO mice (*n* = 6 pairs of mice; each *symbol* represents an individual mouse). *C–E,* titers of TNP-specific IgM, IgG1, or IgG3 in sera of control or cKO mice (*n* = 4) immunized with TNP-KLH in alum (*C*), TNP-Ficoll (*D*), or TNP-LPS (*E*). *Horizontal bars* show the mean value. Data shown are representative of two independent experiments. **, *p* < 0.01.

The transcriptional regulator IκB-ζ can be up-regulated by BCR- or LPS-mediated stimulation of B cells through transcriptional and/or post-transcriptional regulation (24). IκB-ζ-deficient mice exhibit Sjögren's syndrome-like autoimmune disease and abnormal B cell activation (23). However, given that those phenotypes are triggered by epithelial cell death in lacrimal gland, the role of IκB-ζ in B cells remains poorly defined. To better understand the role of IκB-ζ in B cells, we took advantage of Cre-lox technology to generate a B cell-specific deletion of the *Nfkbiz* gene by crossing mice with the *Nfkbiz* flox allele to mice that express the Cre recombinase under the control of the murine Cd79a promoter (Cd79a-Cre, also known as Mb1-Cre). This confirmed that *Nfkbiz* expression in cKO mice was reduced in B cells but not in other immune cells (Fig. 1*A*) (28). These mice appeared healthy and grew without any phenotypic abnormalities (23). Examination of the serum Ig concentration in cKO (Mb-1 Cre;*Nfkbiz^fl^*^/Δ^) mice revealed that levels of IgM, IgG1, IgG2b, IgG3, and IgA were comparable in cKO and control (*Nfkbiz^fl^*^/Δ^) mice (Fig. 1*B*).

Next, we analyzed the role of IκB-ζ in antigen-specific B cell responses by administration of either a TD antigen (TNP-KLH in alum), a TI-2 antigen (TNP-Ficoll), or a TI-1 antigen (TNP-LPS) *in vivo*. In the cases of TNP-KLH and TNP-Ficoll, levels of TNP-specific antibody production were comparable in control and cKO mice (Fig. 1, *C* and *D*). Surprisingly, TNP-specific IgM production induced by TNP-LPS was modestly reduced, and IgG3 production was completely impaired in cKO mice (Fig. 1*E*). These results indicated that the requirement for IκB-ζ is linked specifically to TI-1 antibody responses.

##### Normal B Cell Maturation in IκB-ζ-deficient Mice

**FIGURE 2.**
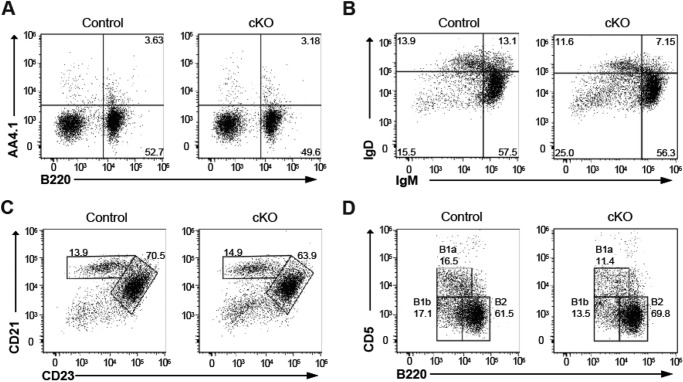
**IκB-ζ is dispensable for B cell maturation.** Flow cytometric analysis of splenocytes (*A–C*) or cells from the peritoneal cavity (*D*) isolated from control or cKO mice. The cells were stained with anti-B220 and anti-AA4.1 antibodies (*A*), anti-B220, anti-IgM, and anti-IgD antibodies (*B*), anti-B220, anti-CD23, and anti-CD21 antibodies (*C*), or anti-B220 and anti-CD5 antibodies (*D*) before analysis by flow cytometry. *Dot plots* were gated on B220^+^ cells (*B–D*). Data shown are representative of four independent experiments.

Given that different subsets of B cells account for different types of antibody responses (29), we next examined whether IκB-ζ deficiency affects the development of subsets of peripheral B cells. The numbers of B220^+^ B cells and B220^+^AA4.1^+^ immature B cells in the spleens of cKO mice were identical to those in control mice (Fig. 2*A*). Likewise, the numbers of sIgM-sIgD^+^ mature B cells, CD21^high^CD23^low^ marginal zone B cells, and CD21^low^CD23^high^ follicular B cells were also the same in the two groups of mice (Fig. 2, *B* and *C*). However, cKO mice had slightly fewer sIgM^+^sIgD^+^ B cells than control mice. These results suggest that IκB-ζ is dispensable for the development of marginal zone and follicular B cells. In addition, subsets of B cells in the peritoneal cavity, such as B1a (B220^low^CD5^hi^), B1b (B220^low^CD5^low^), and B2 (B220^hi^CD5^low^), were equally abundant in cKO and control mice (Fig. 2*D*). These results suggested that B cell maturation does not play a critical role in impairing TI-1 antibody responses in cKO mice.

##### Stimulation of TLR, but Not CD40, Induces IκB-ζ via Post-transcriptional Regulation

**FIGURE 3.**
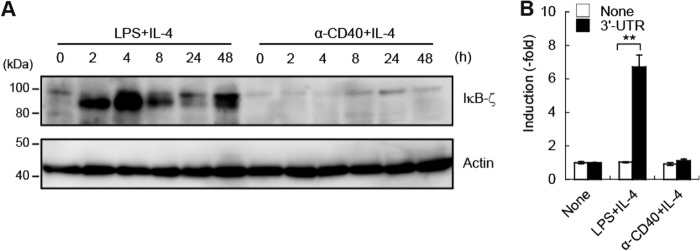
**LPS, but not CD40, induces IκB-ζ expression in B cells.** *A,* immunoblot analysis of IκB-ζ and β-actin in splenic B cells. Purified splenic B cells were stimulated either with 20 μg/ml LPS plus 5 ng/ml IL-4 or with 1 μg/ml anti-CD40 plus 5 ng/ml IL-4 for the time periods indicated. *B,* post-transcriptional activation of IκB-ζ in B cells. CH12F3-2A cells were transfected with pGL4.12-SV40-[luc2CP] (*None*) or pGL4.12-SV40-[luc2CP]-*Nfkbiz*-3′-UTR (3′-UTR) together with phRL-TK-Luc. The cells were stimulated either with 20 μg/ml LPS plus 5 ng/ml IL-4 or with 1 μg/ml anti-CD40 plus 5 ng/ml IL-4 for 4 h before measuring the luciferase activity. Data represent the mean ± S.E. of triplicate samples and are representative of three independent experiments. **, *p* < 0.01.

We next investigated why IκB-ζ deficiency only affects TI-1 antibody responses. Our previous study demonstrated that the induction of IκB-ζ protein following BCR stimulation was weaker than that after TLR stimulation even though the increase in the level of the mRNA that encodes IκB-ζ after BCR stimulation was sufficient to support similar accumulation of IκB-ζ protein (24). The observed differences between transcript abundances and protein levels might thus be attributed to differences in translational regulation after BCR stimulation or TLR stimulation. To examine whether IκB-ζ was induced upon anti-CD40 stimulation, purified splenic B cells were stimulated either with LPS plus IL-4 or with anti-CD40 plus IL-4. As expected, IκB-ζ (85 kDa) was induced only after stimulation with LPS plus IL-4 (Fig. 3*A*). In addition, we found that a 90-kDa modified protein was induced after combined exposure to LPS and IL-4. Although the induction of this modified protein by LPS stimulation was reported previously (18), the nature of the modification remains poorly defined. Given that post-transcriptional regulation of IκB-ζ is activated by TLR/IL-1R but not by stimulation with TNF-α (18, 26), we compared post-transcriptional regulation of IκB-ζ after treatment either with LPS plus IL-4 or with anti-CD40 plus IL-4. Given our previous demonstration that transcriptional activity of the SV40 promoter was dispensable for LPS stimulation (26), we prepared SV40 promoter-driven reporters that expressed an mRNA that included a fusion of the coding sequence of luciferase to the 3′-UTR of the transcript that encodes IκB-ζ. The promoter activity was thus a reliable indicator of the post-transcriptional regulation of IκB-ζ expression. We found that luciferase activity of the IκB-ζ 3′-UTR fusion reporter was activated only upon exposure to LPS plus IL-4 and not after stimulation by anti-CD40 plus IL-4 (Fig. 3*B*). Thus, these results indicated that the 3′-UTR-mediated post-transcriptional regulation of IκB-ζ defines LPS-specific, but not anti-CD40-mediated, induction of IκB-ζ in B cells.

##### Deficiency of IκB-ζ Impairs TLR-mediated in Vitro Antibody Secretion and B Cell Proliferation

**FIGURE 4.**
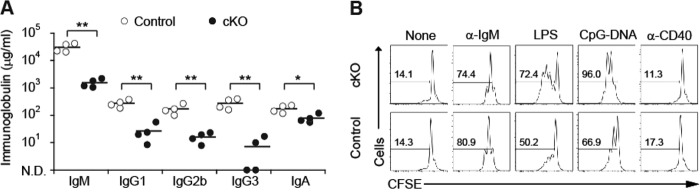
**IκB-ζ is required for Ig secretion and proliferation in response to TLR ligands but not in response to anti-CD40.** *A,* Ig secretion from control or cKO B cells. Purified splenic B cells were stimulated with 20 μg/ml LPS (to determine IgM, IgG2b, and IgG3 levels), 20 μg/ml LPS plus 5 ng/ml IL-4 (to determine IgG1 levels), or 20 μg/ml LPS plus 5 ng/ml IL-4 and 1 ng/ml TGF-β (to determine IgA levels) for 7 days. Concentrations of the indicated Ig in the culture supernatant were measured by ELISA (*n* = 4). *Horizontal bars* show the mean value. *N.D.*, not detected. Data are representative of three independent experiments. *B,* proliferation of control and cKO B cells. Purified splenic B cells were labeled with CFSE and stimulated with 10 μg/ml of the F(ab′)_2_ fragment of anti-mouse IgM (α-*IgM*), 20 μg/ml LPS, 300 nm CpG-ODN, or 1 μg/ml anti-CD40 for 72 h. Cell division was analyzed by flow cytometry. *Numbers* in histograms indicate frequencies of proliferating cells. Data are representative of three independent experiments. *, *p* < 0.05; **, *p* < 0.01.

To establish the mechanistic basis of the defective TI-1 antibody responses in cKO mice, we examined whether purified IκB-ζ-deficient B cells were also impaired in *in vitro* antibody production triggered by LPS stimulation in either the presence or absence of cytokine. After stimulation by exposure to various conditions, we measured levels of Igs secreted into the culture medium. This indicated that IκB-ζ-deficient B cells secreted less IgM, IgG1, IgG2b, IgG3, and IgA than control B cells (Fig. 4*A*). This provided *in vitro* confirmation of the defect of TLR-mediated antibody responses observed in cKO mice. Next, we examined whether the reduced antibody production could be attributed to changes in the proliferation of B cells.

We analyzed the effects of IκB-ζ deficiency on B cell proliferation by monitoring rates of incorporation of the vital dye CFSE. Rates of cell division in control B cells and cKO B cells were indistinguishable following stimulation with either BCR or CD40. However, after stimulation with LPS or CpG-DNA, the rate of division of cKO B cells was considerably less than that of control B cells (Fig. 4*B*). These results demonstrated that IκB-ζ is required for B cell proliferation triggered by TLR stimulation.

##### IκB-ζ Is Essential for TLR-mediated Differentiation of B Cells into Plasma Cells

**FIGURE 5.**
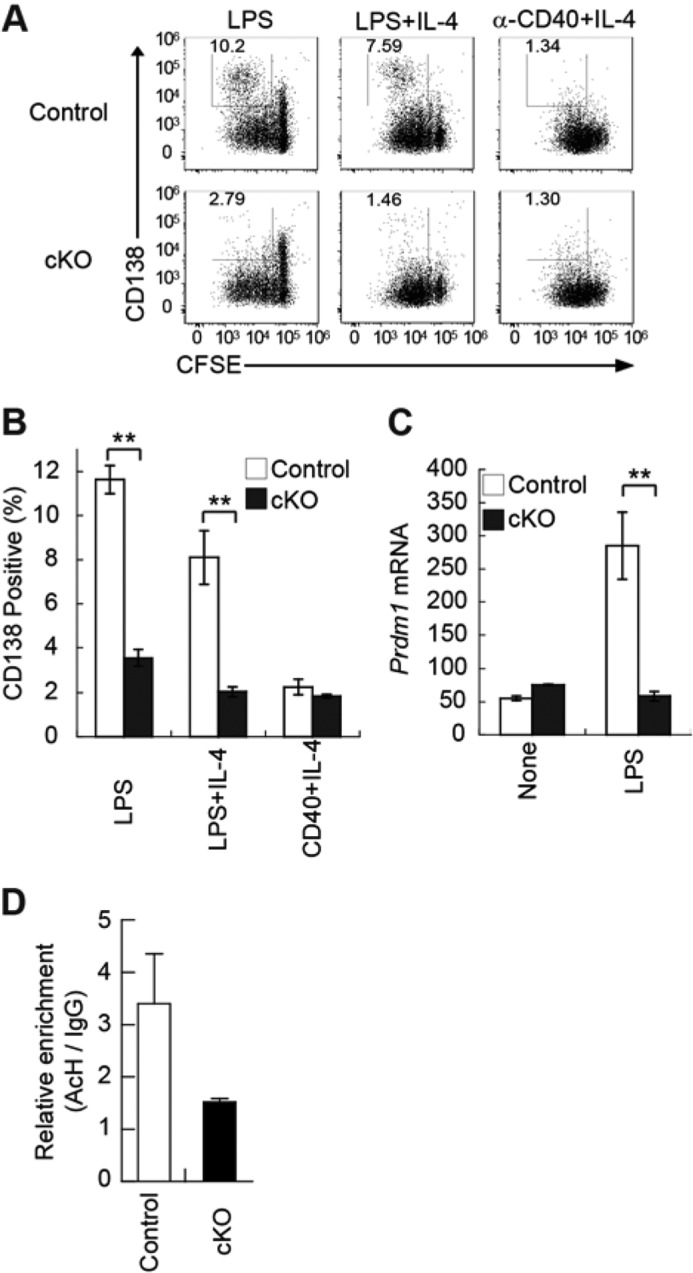
**IκB-ζ-deficient B cells exhibit impaired plasma cell differentiation in response to LPS.** *A* and *B,* plasma cell differentiation of splenic B cells from control or cKO mice. Purified splenic B cells were labeled with CFSE and stimulated for 3 days with 20 μg/ml LPS, 20 μg/ml LPS plus 5 ng/ml IL-4, or 1 μg/ml anti-CD40 (α-CD40) plus 5 ng/ml IL-4. The cells were stained with anti-CD138 antibody and analyzed by flow cytometry. *Numbers* in dot plots indicate the frequencies of CD138^+^ cells in the *boxed area*. Data shown are representative of three independent experiments (*A*). Relative abundances are shown of CD138^+^ cells after exposure to LPS, LPS plus IL-4, or anti-CD40 plus IL-4. Data represent the mean ± S.E. of three independent experiments (*B*). *C,* expression of *Prdm1* mRNA in splenic B cells from control or cKO mice. Purified splenic B cells were stimulated with 20 μg/ml LPS for 72 h. Total RNA was extracted, and *Blimp-1* and *Cd79b* mRNAs were quantified by real time RT-PCR. Copy numbers of *Blimp-1* mRNA per 1000 copies of *Cd79b* mRNA are shown. Data represent the mean ± S.E. of triplicate samples and are representative of three independent experiments. *D,* histone acetylation of the Blimp-1 promoter region in splenic B cells from control or cKO mice. Purified splenic B cells were stimulated with 20 μg/ml LPS plus 5 ng/ml IL-4 for 3 days. Histone acetylation (*AcH*) enrichment was analyzed by a chromatin immunoprecipitation assay performed using antibody against acetyl-histone H3 (Lys-27). Data represent the mean ± S.E. of triplicate samples and are representative of two independent experiments. **, *p* < 0.01.

To analyze whether IκB-ζ regulates the differentiation of plasma cells, purified splenic B cells from control or cKO mice were cultured for 3 days in the presence of either LPS alone, LPS plus IL-4, or anti-CD40 plus IL-4. The results indicated that cKO B cells expressed lower levels of the plasma cell marker CD138 than control B cells (Fig. 5, *A* and *B*). To clarify the molecular mechanism involved, we examined the RNA sequence and found the differences in the levels of *Prdm1* (Coding for Blimp-1), a transcriptional factor required for the differentiation of B cells into plasma cells (Table 3) (30). This indicated that IκB-ζ-deficient B cells failed to express Blimp-1 after stimulation with LPS (Fig. 5*C*). In addition, reduced levels of acetylation of histone H3 in the Blimp-1 promoter region in IκB-ζ-deficient B cells suggested that they contained more active chromatin than unmodified B cells (Fig. 5*D*); however, this difference was not significant (*p* = 0.1865). Thus, IκB-ζ probably controls the differentiation of B cells into plasma cells through inducing Blimp-1 expression.

##### IκB-ζ Is Essential for TLR-mediated CSR

**FIGURE 6.**
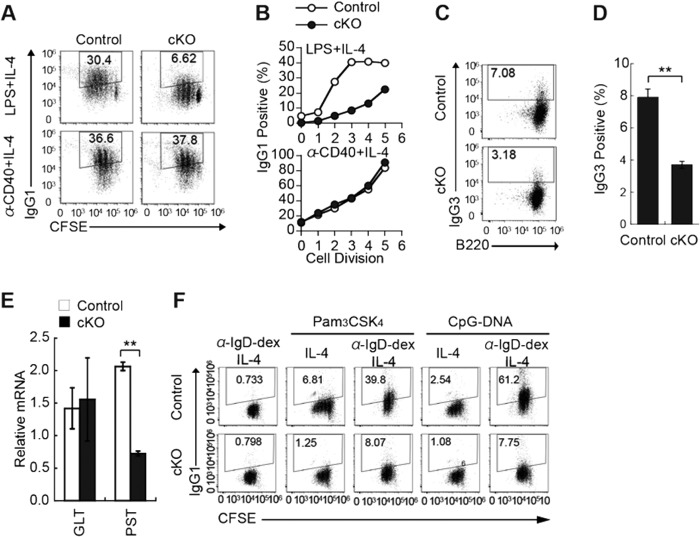
**IκB-ζ-deficient B cells exhibit impaired IgG1 CSR in response to TLR ligands.** *A* and *B,* rates of CSR in splenic B cells from control and cKO mice. Purified splenic B cells were labeled with CFSE and stimulated either with 20 μg/ml LPS plus 5 ng/ml IL-4 or with 1 μg/ml anti-CD40 plus 5 ng/ml IL-4. The cells were stained with anti-IgG1 antibody and analyzed by flow cytometry. *Numbers* in the dot plots indicate the numbers of IgG1^+^ cells in the *boxed area*. Data are representative of four independent experiments (*A*). Frequencies of IgG1^+^ cells in each cell division are shown (*B*). *C,* IgG3 CSR of splenic B cells from control or cKO mice. Purified splenic B cells were labeled with CFSE and stimulated with 20 μg/ml LPS for 3 days. The cells were stained with anti-IgG3 antibody and analyzed by flow cytometry. *D,* frequencies of IgG3^+^ cells in response to LPS. Data shown are the mean ± S.E. of triplicate samples and are representative of three independent experiments. *E,* expression of germ line transcripts and post-recombination transcripts in control or cKO splenic B cells. Purified splenic B cells were stimulated for 3 days with 20 μg/ml LPS plus 5 ng/ml IL-4. Total RNA was extracted, and the germ line I_γ_1-C_γ_1 transcripts and the post-recombination I_μ_-C_γ_1 transcripts were quantified by real time RT-PCR. Expression levels of the germ line transcripts and post-recombination I_μ_-C_γ_1 transcripts (*PST*) were normalized relative to *Cd79b* expression. *F,* purified splenic B cells were labeled with CFSE and stimulated either with 100 ng/ml Pam_3_CSK_4_ plus 5 ng/ml IL-4 or with 1 μm CpG-DNA plus 5 ng/ml IL-4 in the presence or absence of anti-IgD-dextran for 3 days. *Numbers* in dot plots indicate the IgG1^+^ cells in the *boxed area*. Data represent the mean ± S.E. of triplicate samples and are representative of three independent experiments. **, *p* < 0.01.

To assess the effects of IκB-ζ deficiency on CSR, splenic B cells were stimulated either with LPS plus IL-4 or with anti-CD40 plus IL-4 (to induce switching to IgG1). After 3 days of stimulation by LPS plus IL-4, levels of surface IgG1 were lower in cKO B cells than in control B cells (Fig. 6*A*). However, following stimulation with anti-CD40 plus IL-4, levels of surface IgG1 were identical in cKO B cells and control B cells. The impairment of CSR observed in IκB-ζ-deficient B cells could not be attributed to a change in the rate of their proliferation, because there were fewer IgG1-positive B cells in each cell division in populations of cKO B cells than in populations of control B cells (Fig. 6*B*). Similarly, LPS induced fewer IgG3-positive cells when administered to cKO B cells than when administered to control B cells (Fig. 6, *C* and *D*).

To establish what impairs CSR in cKO B cells, we examined whether a reduced rate of CSR in cKO B cells resulted from reduced accumulation of germ line transcripts that encode the intervening heavy chain region and the constant heavy chain region (I_H_-C_H_), which is necessary for CSR (31). Real time quantitative RT-PCR showed that, after stimulation for 3 days with LPS plus IL-4, the abundance of germ line transcripts that encode Iγ1-Cγ1 was similar in cKO B cells and control B cells. In contrast, post-recombination I_μ_-C_γ_1 transcripts, which are generated by CSR, were significantly less abundant in cKO B cells than in control B cells (Fig. 6*E*). Co-engagement of BCR and TLR induces CSR through a noncanonical NF-κB pathway (9). We examined whether IκB-ζ deficiency affects CSR triggered by simultaneous exposure to BCRs and TLRs. Stimulation of control B cells either with anti-IgD-dextran plus Pam3CSK4 (TLR2 ligand) or with CpG-DNA (TLR9 ligand) plus IL-4 caused a strong induction of CSR to IgG1. However, cKO B cells failed to induce CSR (Fig. 6*F*). Taken together, these results indicate that IκB-ζ is essential for the induction of CSR through the co-engagement of BCR and TLR.

##### IκB-ζ Regulates CSR through AID Induction

**FIGURE 7.**
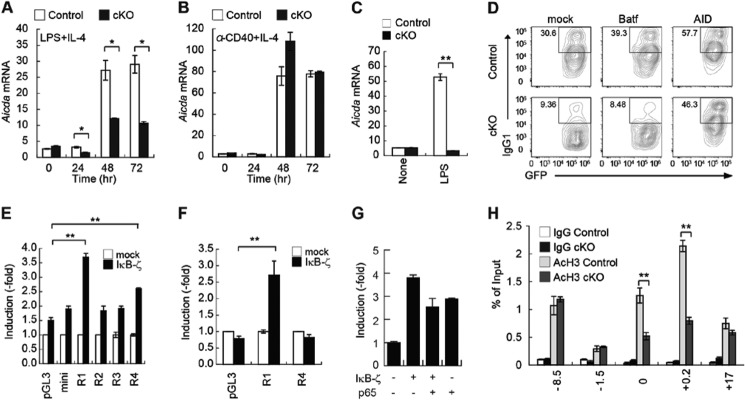
**IκB-ζ promotes CSR through AID induction in response to LPS.** *A* and *B,* levels of *Aicda* mRNA in splenic B cells from control and cKO mice. Purified splenic B cells were stimulated with 20 ng/ml LPS plus 5 μg/ml IL-4 (*A*) or 1 μg/ml anti-CD40 plus 5 ng/ml IL-4 (*B*) for the time periods indicated. Total RNA was extracted, and *Aicda* and *Cd79b* mRNAs were quantified by real time RT-PCR. Copy numbers of *Aicda* mRNA per 1,000 copies of *Cd79b* mRNA are shown. Data shown are the mean ± S.E. of triplicate samples and are representative of three independent experiments. *C,* copy numbers of *Aicda* mRNA per 1,000 copies of *Cd79b* mRNA. Data shown are the mean ± S.E. of triplicate samples and are representative of three independent experiments. *D,* rescue of CSR in splenic B cells from cKO mice. Purified splenic B cells were stimulated with 50 ng/ml anti-IgD-dex for 24 h and retrovirally transduced with pMY-IRES-GFP (mock), pMY-BATF-IRES-GFP (BATF), or pMY-AID-IRES-GFP (*AID*). The cells were then cultured for 3 days in the presence of 20 μg/ml LPS plus 5 ng/ml IL-4. The cells were stained with anti-IgG1 antibody and analyzed by flow cytometry. Contour plots were gated on GFP^+^ cells. *Numbers* in the contour plots indicate the frequencies of IgG1^+^ cells in the *boxed areas*. Data are representative of three independent experiments. *E* and *F,* reporter analysis of *Aicda* promoter in HEK293 cells (*E*) or CH12F3-2A cells (*F*). Cells were transfected with the indicated reporter plasmid harboring the indicated *Aicda* conserved genomic region with or without the IκB-ζ expression plasmid. Data shown are the mean ± S.E. of triplicate samples and are representative of three independent experiments. *G,* HEK293 cells were transfected with a reporter plasmid harboring the genomic region 1 with conserved *Aicda*, with or without IκB-ζ or the plasmid expressing NF-κB subunit p65. The data shown are the mean ± S.D. of duplicate samples and are representative of two independent experiments. *H,* histone acetylation of the AID promoter/enhancer/silencer region in splenic B cells from control or cKO mice. Purified splenic B cells were stimulated with 20 μg/ml LPS plus 5 ng/ml IL-4 for 3 days. Histone acetylation was analyzed by a chromatin immunoprecipitation assay performed using antibody against acetyl-histone H3 (Lys-27). Data represent the mean ± S.E. of triplicate samples and are representative of three independent experiments. *, *p* < 0.05; **, *p* < 0.01.

We next clarified the molecular mechanisms of class switch recombination and found that expression of *Aicda* (coding for AID), the enzyme that induces DNA cleavage in the switch region of the Ig heavy chain locus (so-called CSR), was less in cKO B cells (Table 3) (10, 14). Detection of *Aicda* mRNA by real time quantitative RT-PCR indicated that its abundance peaked within 48–72 h after the stimulation of control B cells induced either by LPS plus IL-4 or by CD40 plus IL-4. However, AID expression in cKO B cells was less than 60% that in control B cells (Fig. 7, *A* and *B*). In addition, LPS failed to induce AID in cKO B cells (Fig. 7*C*). We thus investigated whether the defective CSR in IκB-ζ-deficient B cells resulted from impaired expression of AID. To test this hypothesis, we used retroviral transfection to overexpress AID in cKO B cells and measured isotype switching in response to stimulation by LPS plus IL-4. Consistent with the results shown in Fig. 6*A*, rates of CSR were much lower in cKO B cells transduced with the control retrovirus than in control B cells (Fig. 7*D*). In contrast, overexpression of AID in cKO B cells restored CSR, as shown in control B cells. In addition, retroviral transduction of BATF, which is a key regulator of AID expression (16), did not rescue CSR in cKO B cells. These results suggested that IκB-ζ controls CSR by direct regulation of AID expression.

To assess the role of IκB-ζ in the induction of AID, we examined whether overexpression of IκB-ζ affects the expression of a reporter gene placed under the control of the AID regulatory region. Four regions within the genomic *Aicda* locus are highly conserved among many species (10). These regions are called region 1 (positions −1500 to +101), region 2 (positions +121 to +2221), region 3 (positions +16,278 to +18,378), and region 4 (positions −9224 to −7424). When cells were co-transfected with IκB-ζ and each of the four reporters that contain an AID regulatory region, the region 1-containing AID reporter was most significantly activated in the presence of IκB-ζ in HEK293 cells (Fig. 7*E*). Additionally, the region 4-containing AID reporter was activated in the presence of IκB-ζ in HEK293 cells. To further confirm these findings, we used the B cell line CH12F3-2A and found that the region 1-containing AID reporter, but not the region 4-containing AID reporter, was significantly activated in the presence of IκB-ζ. Therefore, region 1 is more important than region 4 for AID gene expression in the response to IκB-ζ (Fig. 7*F*). It has been reported that IκB-ζ controls NF-κB target gene expression (21). In addition, the NF-κB subunit p65 plays an important role in AID expression (32). We found that overexpression of the NF-κB p65 subunit did not further elevate the activity of the region 1-containing AID reporter in the presence of IκB-ζ, indicating that NF-κB may not have been involved in the effect of IκB-ζ on AID transcription (Fig. 7*G*).

We next analyzed the chromatin structure of the genomic *Aicda* locus in activated B cells. When B cells were activated, histone H3 in the conserved region of the genomic *Aicda* locus was highly acetylated; acetylation of histone H3 is a mark of active chromatin (33). ChIP analysis indicated that histone H3 in the vicinity of the transcriptional starting site (region 1) and +0.2-kb area (region 2) was highly acetylated in control B cells but not in cKO B cells after stimulation with LPS plus IL-4 (Fig. 7*H*). Taken together, these results suggested that IκB-ζ regulates AID expression by controlling access to region 1 and modulating histone acetylation around the transcriptional starting site (region 1).

## DISCUSSION

This study sought to analyze the role of IκB-ζ in B cell antibody responses by characterizing mice deficient in IκB-ζ, specifically in their B cells. In many cases, deficiency of transcriptional regulators impairs both TD and TI antigen responses in precedents (11–13, 15, 16). Here, we have used *in vivo* and *in vitro* assays to show that TLR-mediated TI-1, but not TD or TI-2, antibody responses are impaired in cKO mice. These defects were caused by reduced rates of B cell proliferation, differentiation of B cells into plasma cells, and B cell CSR. This TI-1-specific function of IκB-ζ is assumed to result from TLR-specific induction of IκB-ζ. Induction of IκB-ζ requires threshold levels of transcriptional activation, mRNA stabilization, and translational activation (18, 34). Although TLR4 stimulation satisfies the criteria needed to induce IκB-ζ, stimulation with anti-CD40 antibody failed to initiate post-transcriptional activation of IκB-ζ. In addition, we have shown that BCR stimulation can stabilize IκB-ζ mRNA, although the increase in levels of IκB-ζ protein is less than that triggered by TLR stimulation. This might be caused by reduced rates of translational activation. Mechanistically, the TLR signal molecule MyD88 positively regulates IκB-ζ protein expression (19). However, MyD88-deficient B cells show increased IκB-ζ mRNA expression in response to LPS stimulation, to a level even higher than that of control B cells (data not shown). Thus, the TLR-MyD88 pathway may control the post-transcriptional regulation of IκB-ζ. Therefore, robust induction of IκB-ζ by TLR might define the TI-1-specific function of IκB-ζ.

In the case of CSR, impaired induction of AID contributed substantially to the impairment of CSR in cKO B cells, because levels of germ line transcripts for IgG1 were normal. In fact, retroviral transduction of AID rescued CSR in cKO B cells following stimulation by LPS plus IL-4. Reporter analysis indicated that overexpression of IκB-ζ in HEK293 cells activated AID reporters that contained either region 1 or region 4. However, only the region 1-containing AID reporter was activated in CH12F3-2A cells. Given that CH12F3-2A is a B cell line, IκB-ζ might regulate AID expression by affecting region 1 in B cells. Consistent with this notion, levels of acetylation of histone H3 in region 1 and region 2, but not region 4, were lower in cKO B cells than in control B cells. Given that the expression of the region 2 reporter was not affected by IκB-ζ overexpression, the reduced rate of histone H3 acetylation in region 2 in cKO B cells might not be physiologically relevant. We previously demonstrated that TLR-mediated NF-κB activation was comparable in control and IκB-ζ-deficient B cells (24). Given that inhibition of histone deacetylase activity induces AID expression (35), histone acetylation in the genomic *AICDA* locus might promote AID expression. Furthermore, it has been shown that IκB-ζ and histone deacetylase 5 are co-localized in the nucleus, suggesting that IκB-ζ may function by modulating histone deacetylase 5 activity (36). Taken together, our findings suggest that IκB-ζ would regulate chromatin structure to activate the expression of the gene that encodes AID. Given that TD antibody responses are independent of IκB-ζ, unidentified factors might control AID induction as a substitute for IκB-ζ in TD antibody responses.

IκB-ζ forms a complex with NF-κB and controls NF-κB gene expression (21, 37). A previous study has shown that IκB-ζ positively regulates IL-17A gene expression in combination with RORγt, which is dispensable for NF-κB activation (20, 38). Here, we show that IκB-ζ transcriptional activity the region 1-containing AID reporter is dispensable for NF-κB transcriptional activity.

It is widely thought that TI antibody responses are not as important as TD antibody responses in protecting against infection. However, TI-1 responses are critical for preventing blood-borne infections from evolving into life-threatening conditions (39). In addition, TLR ligands are required for optimal antibody responses against *Streptococcus pneumonia* and after pneumococcal vaccination (40–42). A human patient deficient in IRAK4 (a TLR signaling molecule) presented with a suppressed glycan-specific IgG antibody response after administration of an anti-pneumococcal glycan vaccine (43). Although this vaccine is broadly defined as a TI-2 antigen, it contains a TLR2 ligand and requires IRAK4 for the production of specific antibodies (44). Thus, both BCR and TLR signaling are required for a protective response to this vaccine. We have shown that IκB-ζ is required to induce BCR- and TLR2-dependent antibody responses in mice. In addition, IRAK4 is a key regulator of the activation of IκB-ζ both at the transcriptional and post-transcriptional levels (45). The induction of IκB-ζ in B cells by TLR signaling might play an important role in ensuring the efficacy of an anti-pneumococcal vaccine in humans. Given that IκB-ζ-mediated antibody responses are independent of T cells, obtaining a better understanding of the IκB-ζ-mediated antibody responses might contribute to the development of vaccines for patients with T cell deficiencies, such as individuals with acquired immune deficiency syndrome.

## Footnotes

2 The abbreviations used are:

CSR: class switch recombination
AID: activation-induced cytidine deaminase
BCR: B cell antigen receptor
CFSE: carboxyfluorescein succinimidyl ester
KLH: keyhole limpet hemocyanin
TD: T cell-dependent
TI: T cell-independent
TLR: Toll-like receptor
TNP: 2,4,6-trinitrophenyl
Pam_3_CSK_4_: *S*-[2,3-Bis(palmitoyloxy)-(2-*RS*)-propyl]-*N*-palmitoyl-(*R*)-Cys-(*S*)-Ser-(*S*)-Lys_4_-OH.
